# Microbiological findings, antibiotic resistance rates and prescription practices for older women with recurrent urinary tract infections: a descriptive study alongside a multinational randomized controlled trial (ImpresU)

**DOI:** 10.1093/jacamr/dlag121

**Published:** 2026-07-04

**Authors:** Silje Rebekka Heltveit-Olsen, Nils Grude, Egill Snaebjörnsson Arnljots, Pär-Daniel Sundvall, Ronny Gunnarsson, Anna Kowalczyk, Maciej Godycki-Cwirko, Tamara N Platteel, Wim G Groen, Sara Sofia Lithén, Sofia Sundvall, Nahid Karami, Theo J M Verheij, Cees M P M Hertogh, Morten Lindbæk, Christina Åhrén, Sigurd Høye

**Affiliations:** The Antibiotic Centre for Primary Care, Department of General Practice, Institute of Health and Society, University of Oslo, P.O. Box 1130 Blindern, Oslo 0318, Norway; Department of Medical Microbiology, Vestfold Hospital Trust, Tønsberg, Norway; The Antibiotic Centre for Primary Care, Department of General Practice, Institute of Health and Society, University of Oslo, P.O. Box 1130 Blindern, Oslo 0318, Norway; Department of Medical Microbiology, Vestfold Hospital Trust, Tønsberg, Norway; General Practice/Family Medicine, School of Public Health and Community Medicine, Institute of Medicine, Sahlgrenska Academy, University of Gothenburg, Box 453, Gothenburg SE-405 30, Sweden; FoUUI-Centrum Södra Älvsborg, Research, Education, Development & Innovation, Primary Health Care, Region Västra Götaland, Sweden, Sven Eriksonsplatsen 4, Borås SE-503 38, Sweden; Närhälsan Heimdal Primary Health Care Centre, Stengärdsgatan 22, Borås 50334, Sverige; General Practice/Family Medicine, School of Public Health and Community Medicine, Institute of Medicine, Sahlgrenska Academy, University of Gothenburg, Box 453, Gothenburg SE-405 30, Sweden; Allmänmedicinskt Centrum, Research, Education, Development & Innovation, Primary Health Care, Region Västra Götaland, Sweden, Guldhedsgatan 5A, Gothenburg SE-413 20, Sweden; Centrum for Antibiotic Resistance Research (CARe), University of Gothenburg, Gothenburg, Sweden; Närhälsan Sandared Primary Health Care Centre, Strandvägen 11, Sandared 518 32, Sweden; General Practice/Family Medicine, School of Public Health and Community Medicine, Institute of Medicine, Sahlgrenska Academy, University of Gothenburg, Box 453, Gothenburg SE-405 30, Sweden; Allmänmedicinskt Centrum, Research, Education, Development & Innovation, Primary Health Care, Region Västra Götaland, Sweden, Guldhedsgatan 5A, Gothenburg SE-413 20, Sweden; Centrum for Antibiotic Resistance Research (CARe), University of Gothenburg, Gothenburg, Sweden; Cairns Clinical School, College of Medicine and Dentistry, James Cook University, PO Box 6811, Cairns, QLD 4870, Australia; Centre for Family and Community Medicine, the Faculty of Health Sciences, The Medical University of Lodz, Lodz 90-419, Poland; Centre for Family and Community Medicine, the Faculty of Health Sciences, The Medical University of Lodz, Lodz 90-419, Poland; Julius Center for Health Sciences and Primary Care, University Medical Center Utrecht, Universiteitsweg 100, Utrecht 3584 CG, The Netherlands; Department of Medicine for Older People, Amsterdam UMC, Vrije Universiteit Amsterdam, De Boelelaan 1117, Amsterdam 1081 HV, The Netherlands; Amsterdam Public Health Research Institute, Aging & Later Life, Amsterdam, The Netherlands; The Antibiotic Centre for Primary Care, Department of General Practice, Institute of Health and Society, University of Oslo, P.O. Box 1130 Blindern, Oslo 0318, Norway; FoUUI-Centrum Södra Älvsborg, Research, Education, Development & Innovation, Primary Health Care, Region Västra Götaland, Sweden, Sven Eriksonsplatsen 4, Borås SE-503 38, Sweden; Centrum for Antibiotic Resistance Research (CARe), University of Gothenburg, Gothenburg, Sweden; Institute of Biomedicine, Department of Infectious Diseases, University of Gothenburg, Gothenburg, Sweden; Sahlgrenska University Hospital, Department of Clinical Microbiology, Region Västra Götaland, Gothenburg 41345, Sweden; Julius Center for Health Sciences and Primary Care, University Medical Center Utrecht, Universiteitsweg 100, Utrecht 3584 CG, The Netherlands; Department of Medicine for Older People, Amsterdam UMC, Vrije Universiteit Amsterdam, De Boelelaan 1117, Amsterdam 1081 HV, The Netherlands; Amsterdam Public Health Research Institute, Aging & Later Life, Amsterdam, The Netherlands; The Antibiotic Centre for Primary Care, Department of General Practice, Institute of Health and Society, University of Oslo, P.O. Box 1130 Blindern, Oslo 0318, Norway; Centrum for Antibiotic Resistance Research (CARe), University of Gothenburg, Gothenburg, Sweden; Institute of Biomedicine, Department of Infectious Diseases, University of Gothenburg, Gothenburg, Sweden; Swedish Strategic Program against Antimicrobial Resistance (Strama), Region Västra Götaland, Gothenburg, Sweden; The Antibiotic Centre for Primary Care, Department of General Practice, Institute of Health and Society, University of Oslo, P.O. Box 1130 Blindern, Oslo 0318, Norway

## Abstract

**Objectives:**

To describe microbiological findings during asymptomatic and symptomatic phases in older women with recurrent urinary tract infections (rUTIs), to evaluate whether antimicrobial resistance patterns of *Escherichia coli* among methenamine hippurate users differ from untreated controls, and to explore prescription patterns of UTI antibiotics.

**Methods:**

This was a descriptive study alongside a multinational randomized controlled trial (ImpresU). Women aged ≥70 years with rUTIs were recruited from general practice in Norway, Sweden, Poland and the Netherlands between December 2019 and June 2022. Participants were randomized to methenamine hippurate 1 g or placebo twice daily for 6 months, followed by a 6 month follow-up period. Urinary samples were collected at baseline (asymptomatic) and during acute UTIs.

**Results:**

We included 281 women. A dominating uropathogen ≥10^5 ^cfu/mL was detected in 29% of the baseline urine samples. Of the 178 urinary cultures taken at acute UTIs, 163 (92%) had bacterial growth, of which a minority (10%) were reported as mixed flora. *E. coli* was most frequently detected both at baseline and in acute UTIs (present in any concentration in 32% versus 60% of urine samples), with highest resistance to amoxicillin (36% versus 43%), trimethoprim (16% versus 22%) and amoxicillin/clavulanate (12% versus 16%). ESBL-producing *E. coli* at baseline were rare (4.1%). Phylogenetic subgroup B2 was the most frequent *E. coli* phylotype at baseline (52%). No relevant differences in antimicrobial resistance rates were found between methenamine hippurate users and controls. Narrow-spectrum antibiotics were widely prescribed, and guideline adherence was high.

**Conclusions:**

Physicians should be aware that 29% of older women with rUTIs in the community may have significant bacterial findings in their urine during asymptomatic phases.

## Introduction

Urinary tract infections (UTIs) are the most prevalent infections in humans, with older, postmenopausal women being particularly susceptible for recurrent episodes.^1^ Significant bacteriuria in the absence of symptoms, referred to as asymptomatic bacteriuria (ABU), does not warrant treatment and is common among older adults. The prevalence of ABU has been reported to be 2.8%–8.6% in healthy postmenopausal women (50–70 years) increasing to 11%–16% in older women (≥70 years) in the community, and to 25%–50% in long-term care facilities.^2^ However, the prevalence of ABU in the subgroup of older women suffering from recurrent UTIs is poorly explored. Uropathogenic *Escherichia coli* (UPEC) is the leading cause of UTIs in humans, reported to account for as much as 75% of uncomplicated UTIs and 65% of complicated UTIs.^3,4^ Evidence suggests that repeated courses of antibiotics may contribute to increased rates of UTIs, with recurrences frequently attributed to relapses of the same strain of *E. coli.*^5–8^  *E. coli* has an extensive phylogenetic substructure. According to Clearmont typing and classification there are currently nine recognized main phylotypes of *E. coli* with eight belonging to the classic *E. coli* strains (A, B1, B2, C, D, E, F, G) and one corresponding to the cryptic *E. coli* lineage clade I.^9–12^ Strains causing extra-intestinal infections, such as UTIs, compared with commensals, more frequently belong to B2 or D.^13–15^ Phylogroup B2 is shown to have most virulence factors,^16^ and antibiotic exposure is shown to increase the proportion of bacteria expressing antibiotic resistance genes.

The complexity of selection for antimicrobial resistance (AMR) and increasing MDR in uropathogens^17^ underscores the importance of promoting appropriate antibiotic use and exploring effective non-antibiotic prophylactic treatment options for recurrent UTIs (rUTIs). The availability of antibiotics and guideline recommendations vary slightly between European countries. While pivmecillinam is an important first-choice treatment for UTIs in Norway and Sweden, it is not used in Poland and the Netherlands. Conversely, fosfomycin is widely used in Poland and the Netherlands but not included in the guidelines in Norway and Sweden.^18–21^

The ImpresU project set out to explore the prophylactic effect of the urinary antiseptic drug methenamine hippurate on rUTIs (two or more antibiotic courses for UTIs in the last 6 months and/or   three or more in the last 12 months) in women aged ≥70 years in primary healthcare settings.^22^ Alongside this randomized controlled trial (RCT), urinary samples were collected from all participants at baseline (asymptomatic) and from subsequent episodes of acute UTIs according to clinical practice. Information on antibiotic treatment choices of the clinicians was also collected. This presented us with a unique opportunity to: (i) explore microbiological findings in the urine of this key demographic of older women with rUTI in both asymptomatic and symptomatic phases; (ii) to evaluate whether antimicrobial resistance patterns of *E. coli* during and after preventive use of methenamine hippurate differ from the resistance pattern in untreated controls; and (iii) to explore prescription patterns of UTI antibiotics in four diverse European countries.

## Patients and methods

### Study design

The RCT was part of the project ‘Improving rational prescribing for urinary tract infections in frail elderly’ (ImpresU)—a collaboration between researchers in Norway, Sweden, Poland and the Netherlands.^22,23^ Participants were recruited for the RCT from December 2019 to June 2022, and the data collection was completed by end of June 2023. The trial was registered at clinicaltrials.gov (NCT04077580) and with EudraCT (2018-002235-15).

### Participants and setting

Women aged ≥70 years with a history of rUTIs (two or more antibiotic treatments for UTIs the last 6 months and/or three UTIs in the last 12 months prior to inclusion) were recruited from general practice in Norway, Sweden, Poland and the Netherlands.

### Procedures

#### Collection of urinary samples

Urinary samples were collected from all participants at baseline when the participants were asymptomatic. Urine samples were also obtained during symptomatic episodes of acute UTIs treated with antibiotics during the 12 month study period—if requested by the clinician according to routine clinical management in the respective country. Procedures for management of the urine samples were consolidated in a separate microbiology plan (see Supplementary methods; available as Supplementary data at *JAC-AMR* Online). Preceding any study-related procedures, informed consents were obtained from all participants. Participants were instructed to take a morning mid-stream urine sample on the day of the inclusion visit and to keep the urine sample refrigerated if obtained more than 2 h before the visit.

#### Microbiological analyses

Baseline urine samples were sent to designated study laboratories in each participating country for analysis. Species identification was performed according to routine clinical microbiology practice, and antibiotic sensitivity testing was performed according to the disc diffusion method and breakpoints according to EUCAST (www.eucast.org) at the time.^24^ All samples from acute UTIs during the study period were managed by the regular health services following clinical practice. As resistance pattern analyses were performed according to the local laboratory routines in each country, no pre-defined list of antibiotics was used for these samples.

#### Analysis of phylogenetic subgroups and resistance pattern of baseline E. coli isolates

Isolates of *E. coli*, regardless of concentration (also if part of a mixed culture), were frozen and shipped to Sahlgrenska University Hospital (Gothenburg, Sweden) for further examination. Resistance pattern analyses for 11 pre-selected antibiotics were performed using disc diffusion in accordance with the EUCAST recommendations.^24^  *E. coli* isolates resistant to one of two indicator cephalosporins (cefotaxime, ceftazidime) were tested for ESBL production using double disc diffusion assay.^25^ Phylogenetic subtyping (subgroups A, B1, B2, C, D, E, F, G and clade I) was performed using Quadruplex PCR.^26^

### Statistical analysis

All descriptive statistics and regression analyses were performed using Stata (version 18). A two-sample test of proportions was used to test the equality of proportions between the methenamine hippurate group and the placebo control group at baseline and at acute UTIs. The level of significance was set to be 0.05.

To compare proportions of antibiotic resistance in *E. coli* isolates from acute UTIs between the methenamine hippurate group and the placebo group adjusted for baseline resistance, we used logistic regression. As data for acute UTIs were clustered, with some subjects having more than one observation/episode, we used mixed-effects logistic regression with subject as a random effect and adjustment for baseline values as fixed effects. Group allocation was added as a fixed effect.

For the antibiotic amoxicillin/clavulanate, mixed-effects logistic regression was not possible due to a low frequency of outcome in one allocation group. To provide more reliable statistical inference with a small-sample dataset with binary outcome, we assessed the effect of allocation on resistance using the exact logistic regression model.^27^

## Results

### Study population

The ImpresU study population consisted of 289 women aged ≥70 years with a history of rUTIs. In this report, we excluded eight women due to incomplete data for the baseline urine tests, leaving us with 281 women for analysis. A full table of baseline characteristics for the study population is already published.^23^ Mean age of the women was 78 years (SD 5.6), with median age 78 and IQR 74–82. The participants had experienced 978 UTI episodes in total in the last 12 months before inclusion in the RCT (mean number of UTIs per participant 3.5, SD 1.5) with 566 of the UTIs occurring in the last 6 months before inclusion (mean number of UTIs per participant 2.0, SD 1.2). Recurrence rate prior to inclusion did not differ significantly between the treatment groups. The inclusion by country was 100 (Sweden), 95 (the Netherlands), 76 (Norway) and 10 (Poland).

### Baseline urine samples

#### Baseline urine cultures

Of the 281 baseline cultures, 80 (29%) urine samples contained at least one dominant bacterial strain in a concentration of ≥10^5 ^cfu/mL. Of these, 57 (20% of all baseline urine samples) were *E. coli* (Table 1). Any growth of *E. coli* was detected in 91 of the urine samples (32%), making it the most frequently detected strain.

**Table 1.** Uropathogens detected in baseline urine samples

Microbe	Any growth in baseline urine samples (*N* = 281), *n* (%)	Amount ≥10^5^ cfu/mL (*N* = 281), *n* (%)
*E. coli*	91 (32)	57 (20)
*Enterococcus* spp.	20 (7.1)	13 (4.6)
*Klebsiella* spp.	10 (3.6)	8 (2.8)
*Proteus* spp.	7 (2.5)	2 (0.71)
Coagulase-negative *Staphylococcus*	5 (1.8)	1 (0.36)
*Aerococcus* spp.	4 (1.4)	2 (0.71)
*Streptococcus agalactiae* (group B strep)	4 (1.4)	2 (0.71)
*Morganella* spp.	2 (0.71)	1 (0.36)
*Enterobacter* spp.	1 (0.36)	1 (0.36)
Group C/G *Streptococcus*	2 (0.71)	0 (0.0)
*Hafnia alvei*	1 (0.36)	1 (0.36)
*Acinetobacter lwoffii*	1 (0.36)	0 (0.0)

#### Phylogenetic subgroups of E. coli at baseline

Phylogenetic subgroups in 73 of 91 (80%) *E. coli* isolates from the baseline urine cultures were determined. Phylogenetic subtype B2 was predominant (38, 52%), equally distributed across treatment groups (Table S1). A total of 47 (64%) of the *E. coli* isolates were classified within phylogenetic subgroups B or D. Only three (4.1%) of the *E. coli* strains were found to be ESBL-producing, with two belonging to subgroup B2 and one to subgroup D (Table 2).

**Table 2.** Phylogenetic subgroups of *E. coli* and ESBL production in baseline urine samples

Phylogenetic subgroup	Total (*N* = 73), *n* (%)	ESBL+ (*N* = 73), *n* (%)
A	5 (6.8)	0 (0.0)
B1	10 (14)	0 (0.0)
B2	38 (52)	2 (2.7)
C	3 (4.1)	0 (0.0)
D	9 (12)	1 (1.4)
E	2 (2.7)	0 (0.0)
F	5 (6.8)	0 (0.0)
Unknown	1 (1.4)	0 (0.0)
Total	73 (100)	3 (4.1)

#### Resistance patterns for E. coli in baseline urine samples

All 73 *E. coli* isolates were tested for resistance pattern to 11 pre-selected antibiotics. We found resistance to amoxicillin in 26 (36%) isolates, to trimethoprim in 12 (16%) isolates and to amoxicillin/clavulanate in 9 (12%) isolates (Table 3). Resistance to the recommended or first-choice UTI antibiotics in the participating countries was in all cases found to be low, with five isolates resistant to pivmecillinam (5.5%), three isolates with resistance to fosfomycin (4.1%) and one isolate with resistance to nitrofurantoin (1.4%). Differences in proportions of resistance at baseline by allocation are presented in Table S2.

**Table 3.** Resistance patterns for *E. coli* in baseline urine samples

Resistance pattern *E. coli*, baseline urine	Total (*N* = 73)	
	Resistant, *n*	Resistant, %
Amoxicillin	26	36
Trimethoprim	12	16
Amoxicillin/clavulanate	9	12
Ciprofloxacin	5	6.8
Pivmecillinam	4	5.5
Fosfomycin	3	4.1
Cefotaxime	3	4.1
Gentamicin	3	4.1
Ceftazidime	1	1.4
Nitrofurantoin	1	1.4
Meropenem	0	0.0

### Acute UTIs

#### Urine cultures

There were 399 episodes of acute, symptomatic UTIs recorded during the study. In 178 (45%) of the episodes the urine was sent for culture analysis. In Norway, culture was done in 77% (73/95), in Sweden in 42% (61/146), in Poland in 33% (5/15), and in the Netherlands in 27% (39/143) of the UTI episodes. Of the 178 urinary cultures, 163 (92%) samples had bacterial growth, of which a minority (10%) were reported as mixed flora. The most frequent microbe found was *E. coli*, which was present in 107 (60%) of the urinary cultures (Table 4).

**Table 4.** Culture results in acute urinary tract infections

Microbe	Reported growth (*N*= 178), *n* (%)	Reported ≥10^5 ^cfu/mL or ‘significant amount’ (*N* = 178), *n* (%)
*E. coli*	107 (60)	86 (48)
*Klebsiella* spp.	24 (14)	21 (12)
*Proteus* spp.	7 (3.9)	3 (1.7)
*Enterococcus* spp.	7 (3.9)	5 (2.8)
*Aerococcus* spp.	4 (2.2)	3 (1.7)
*Citrobacter* spp.	3 (1.7)	2 (1.1)
*Pseudomonas aeruginosa*	2 (1.1)	0 (0.0)
*Enterobacter* spp.	1 (0.56)	1 (0.56)
*Serratia marcescens*	1 (0.56)	1 (0.56)
Coagulase-negative *Staphylococcus*	1 (0.56)	—
Mixed flora	18 (10)	—

#### Antibiotic resistance patterns for E. coli during acute UTIs

In total, 107 *E. coli* isolates were tested for resistance patterns in local laboratories during the study (Table 5). We collected only the resistance pattern that was released to the clinician from the laboratory. We found resistance to amoxicillin in 19/44 tested isolates (43%), to amoxicillin/clavulanate in 9/55 tested isolates (16%) and to trimethoprim in 19/87 tested isolates (22%). Resistance to first-choice or recommended UTI antibiotics was again found to be low, with 4/84 tested isolates (4.8%) resistant to nitrofurantoin and no resistance found to pivmecillinam (0/73) or fosfomycin (0/11). When comparing the differences in proportions of *E. coli* resistance in acute UTIs adjusted for baseline differences in resistance we did not find any significant differences between the women receiving methenamine hippurate treatment and the placebo group.

**Table 5.** Resistance patterns of *E. coli* in acute urinary tract infections

Acute UTIs with *E. coli*	Total (*N* = 107)			Treatment			Control			*P* value of difference in proportions of resistance between treatment groups	*P* value of difference in proportions of resistance between treatment groups, adjusted for baseline resistance
				Methenamine hippurate (*N* = 57)			Placebo (*N* = 50)				
Resistance pattern	Tested, *n*	Resistant, *n*	Resistant, %	Tested, *n*	Resistant, *n*	Resistant, %	Tested, *n*	Resistant, *n*	Resistant, %		
Nitrofurantoin	84	4	4.8	42	2	4.7	42	2	4.7	1.000	0.929
Pivmecillinam	73	0	0	36	0	0	37	0	0	No resistance found	No resistance in acute UTIs
Fosfomycin	11	0	0	8	0	0	3	0	0	No resistance found	No resistance in acute UTIs
Trimethoprim	87	19	22	47	14	30	40	5	13	0.052	0.938
Ciprofloxacin	55	4	7.3	30	3	10	25	1	4.0	0.394	0.996
Amoxicillin/clavulanate	55	9	16	30	8	27	25	1	4.0	0.024	0.462
Amoxicillin	44	19	43	25	13	52	19	6	32	0.176	0.998

#### Antibiotics

Of the 399 episodes of acute UTIs, 146 episodes were treated with antibiotics in Sweden (37%), 143 in the Netherlands (36%), 95 in Norway (24%) and 15 in Poland (3.8%). Overall, the most frequently prescribed antibiotics were nitrofurantoin (35%), pivmecillinam (32%) and fosfomycin (13%)—with some differences between countries (Table S3). According to local guidelines, first-choice or recommended UTI treatments were prescribed in 89% of cases in Norway and Sweden, 86% in the Netherlands and 67% in Poland.^18–21^

## Discussion

### Main results

Of 281 collected baseline samples from asymptomatic older women with rUTIs, 29% contained significant bacteriuria in a concentration ≥10^5 ^cfu/mL. Of the 178 urinary cultures collected at acute UTIs, 163 (92%) had bacterial growth, of which a minority (10%) were reported as mixed flora. *E. coli* dominated both in baseline cultures and in acute UTIs (growth detected in 32% versus 60%), with highest resistance to amoxicillin (36% versus 43%), trimethoprim (16% versus 22%), and amoxicillin/clavulanate (12% versus 16%). Phylogenetic subgroup B2 was the most frequent *E. coli* phylotype at baseline (52%), and 4.1% of the *E. coli* strains were ESBL-producing. When comparing the proportions of antibiotic resistance of *E. coli* isolates from acute UTIs adjusted for baseline differences we did not find any significant differences between the methenamine hippurate and the placebo group. Across all four countries, the most frequently prescribed antibiotics for acute UTIs in this population were nitrofurantoin (35%), pivmecillinam (32%) and fosfomycin (13%), and guideline adherence was notably high.

### Strengths and limitations

In the setting of a large international RCT, we were provided with the opportunity to explore urine findings in asymptomatic and symptomatic older women with rUTIs in detail. The design also allowed us to report antibiotic treatment strategies for rUTIs across four diverse European countries and compare resistance patterns of *E. coli* of the women treated with methenamine hippurate with those of the untreated controls in this key demographic of older women with rUTIs.

However, the design also had some limitations. While we were able to describe the prevalence of bacteriuria ≥10^5 ^cfu/mL in urine in the asymptomatic phase, we were not able to repeat the analysis to establish the prevalence of asymptomatic bacteriuria in this population according to the ABU definition, requiring findings in repeated sampling.^2^ All baseline urine samples were cultured, and *E. coli* isolates were frozen. However, while Norwegian and Swedish guidelines recommended urine cultures for acute UTIs for women with rUTIs, the Dutch and Polish GP guidelines did not. As a result, only about half of the acute UTIs during the study had available culture results. For the baseline analysis of resistance patterns, all *E. coli* isolates were tested for the same pre-defined set of 11 antibiotics, ESBL production and phylogenetic subtype in the same laboratory. However, in this large international long-term clinical trial it was not feasible to use only one microbiology lab for resistance pattern analyses beyond the baseline urine samples. Even though all laboratories followed the EUCAST recommendations and guidelines, local procedures for which antibiotics to test and report varied slightly. We only collected resistance pattern data released to the clinician, which mainly consisted of oral antibiotics. This resulted in too few observations for some of the tested antibiotics at acute UTIs to make meaningful statistical comparisons of proportions of resistance between treatment groups in the RCT. For the same reason, it was not feasible to obtain data on ESBL production or *E. coli* phylotype for the acute UTIs in this study for comparison with the baseline result. Finally, this study was not powered with means to detect differences in resistance patterns between the two treatment groups. Urine samples were sent for culture and resistance pattern analysis in only 45% of the recorded episodes of acute UTIs in this study, and our results could potentially lack representation for the full study population. This could, along with any variations in the antibiograms released to the clinician, contribute to an over- or underestimation of resistance rates. We did not confirm whether *E. coli* detected in the baseline urine samples were of the same strains causing the acute infections.

### Comparison with literature

The prevalence of ABU in women ≥70 years in community dwellings has previously been reported to be 11%–16%.^2,28,29^ We found that as many as 29% of the 281 women aged ≥70 years with rUTIs had bacteriuria ≥10^5 ^cfu/mL in the asymptomatic phase. A higher proportion of ABU among postmenopausal women with rUTIs has also been reported by Beerepoot *et al.* (48%).^30^ Similar to our study, no subsequent sample after 2 weeks was obtained and a single-voided specimen at baseline was used to indicate ABU.^30^ The higher prevalence of ABU in women with rUTI compared with the reported prevalence in the general population of older women might to some extent be explained by the fact that both studies collected single-voided samples instead of using two consecutive samples in accordance with the ABU definition (≥10^5^ cfu/mL in two consecutive voided urine specimens obtained 2 weeks apart).^2^ The diagnostic criteria for ABU in women are based on evidence that 10%–60% of women do not have persistent ABU in the second urine sample after an initial positive culture. The percentage varies with varying populations, and there are also identified populations where the prevalence of ABU is known to be higher, i.e. older institutionalized persons.^31,32^ However, there are no studies comparing two consecutive asymptomatic urine samples specifically in the at-risk population of older women in community dwellings diagnosed with rUTIs, meaning that the true rate of persistence of ABU from the first to the second sample is not known for this population. At the same time, it is indicated that repeated antibiotic courses might increase the frequency of UTIs and that recurrences of UTIs often are caused by relapses of the same strain of *E. coli*^5–8^—indicating a possible relapse of a colonizing flora. Like Beerepoot *et al.*, we therefore find it possible that our result reflects a higher prevalence of ABU in older women aged ≥70 years with rUTIs than in the general female population aged ≥70 years in the community, despite detection in a single-voided sample. However, in a clinical setting it is important for physicians to be aware of the prevalence of significant bacteriuria in a single-voided sample from this population to facilitate appropriate assessments.

The most prevalent microbe in both baseline urine and in symptomatic UTIs was *E. coli* followed by other Enterobacterale*s* and *Enterococcus* spp., and most resistance was detected for amoxicillin, amoxicillin/clavulanate and trimethoprim. This finding is in accordance with previous reports.^33–36^ However, while the ALTAR study demonstrated that antimicrobial resistance in *E. coli* grew proportionally higher in women receiving low-dose prophylactic antibiotics compared with women treated with methenamine hippurate,^37^ no larger study has compared antimicrobial resistance between women receiving methenamine hippurate and untreated controls. When comparing the proportions of *E. coli* antibiotic resistance at acute UTIs, adjusted for baseline resistance in our study, no significant differences were observed between women receiving methenamine hippurate treatment and those receiving placebo. This finding suggests that AMR development in *E. coli* isolates to the most common urinary antibiotics is comparable to untreated controls and therefore not influenced by the preventive use of this urinary antiseptic drug. However, the RCT was not powered or designed with means to detect such a difference, and the result must be interpreted with caution.

The prevalence of UTIs caused by ESBL-producing *E. coli* is on the rise in both hospitalized and non-hospitalized patients, and rUTIs are found to be a risk factor for ESBL selection.^38,39^ We found that 4.1% of the baseline *E. coli* isolates from our population of older women with rUTIs were ESBL-producing. The prevalence of ESBL-producing Enterobacterale*s* in asymptomatic patients has been explored in several at-risk patient groups, such as pregnant women^40,41^ and renal transplant patients,^42^ as well as during symptomatic UTIs in patients with rUTIs.^43^ However, we were unable to find previous evidence of the prevalence of ESBL-producing *E. coli* in asymptomatic phases for older women in community dwellings diagnosed with rUTIs. Our result is consistent with the Norwegian prevalence of 3.9% ESBL producers in *E. coli* urine isolates.^36^ We also found that ESBL production in this population was linked to *E. coli* phylogroups B2 and D, as previously reported by us and others.^8,16^ The most prevalent *E. coli* phylotype in baseline urine was B2, and phylogroups B2 and D accounted for as many as 64% of the *E. coli* isolates. This finding is in accordance with previous findings in urine from an asymptomatic hospitalized geriatric population ≥75 years of age.^44^

### Clinical implications and further research

The discovery that nearly one-third of older women with rUTIs in the community may have bacteriuria in asymptomatic phases impacts interpretations of diagnostic tests and treatment decisions for acute UTIs for this population. This finding supports that healthcare providers should focus on UTI symptoms rather than relying on urine cultures when assessing suspected UTIs in this demographic. Our results did not demonstrate any significant differences in *E. coli* resistance rates between the women in the methenamine hippurate group and the placebo group, suggesting that the resistance rates were not influenced by preventive use of the drug. However, this finding must be interpreted with caution due to limitations of the dataset.

### Conclusions

Physicians should be aware that 29% of older women with rUTIs in the community may have significant bacterial findings in their urine during asymptomatic phases.

## Supplementary Material

dlag121_Supplementary_Data

## Data sharing

Relevant anonymized data can be made available upon reasonable request.

## Supplementary data

Supplementary methods and Tables S1 to S3 are available as Supplementary data at *JAC-AMR* Online.
