# Supplementary material for: Microbiological findings, antibiotic resistance rates and prescription practices for older women with recurrent urinary tract infections: a descriptive study alongside a multinational randomized controlled trial (ImpresU)
Source: JAC Antimicrob Resist. 2026 Jul 4;8(4):dlag121. doi: 10.1093/jacamr/dlag121 (PMC13332803; doi:10.1093/jacamr/dlag121)
Supplement: dlag121_Supplementary_Data [file dlag121_supplementary_data.docx]

**Supplementary data.**

**Procedure for urinary samples in WP3**

Procedure at the GP office

1. Use a sterile urine specimen container.

2. The urine sample should be midstream, and preferably morning urine. If it is not feasible to obtain morning urine, the urine sample should be obtained at least 3-4 hours after last urination.

3. The patient should be instructed how to take a midstream urine sample.

4. The patient should be informed that if the urine sample is obtained more than 2 hours before it is analyzed, the container should be placed in a refrigerator at a temperature of 0-5 °C.

5. For urinary cultures, use a boric acid transport medium. Alternatively, a cooled transportation box at a temperature of 0-5 °C can be used.

Procedures at microbiology laboratory

All participating laboratories are following recommendations of the European Committee on Antimicrobial Susceptibility Testing (EUCAST).

1. Inclusion urinary culture

- Each laboratory will follow internal guidelines regarding culturing and susceptibility patterns according to European guidelines (EUCAST).
- MIC/sensitivity follows testing breakpoint tables for interpretation of MIC and zone diameter as recommended by EUCAST.
- For the inclusion urinary culture, all isolates of *E. coli* regardless of concentration will be examined for urease activity and frozen and shipped to Sahlgrenska University Hospital (Gothenburg) for further examination of phylogenetic subgroups.

1. Urinary cultures taken during an episode of acute UTI

- Each laboratory will follow internal guidelines regarding culturing and susceptibility patterns according to European guidelines (EUCAST).
- MIC/sensitivity follows testing breakpoint tables for interpretation of MIC and zone diameter as recommended by EUCAST.

Table S1: Phylogenetic subgroup of *E. coli* in baseline urine by treatment allocation

| Phylogroup of *E coli* | A | B1 | B2 | C | D | E | F | Not  known | Total  (n=73) |
| --- | --- | --- | --- | --- | --- | --- | --- | --- | --- |
| Treatment group |  |  |  |  |  |  |  |  |  |
| Methenamine hippurate | 0 | 5 | 18 | 2 | 6 | 1 | 3 | 1 | 36 |
| Placebo tablets | 5 | 5 | 20 | 1 | 3 | 1 | 2 | 0 | 37 |

Table S2: Resistance pattern *E. coli* in baseline urine by allocation

| Resistance pattern *E coli,* baseline urine | Methenamine hippurate  N=36 | | | Placebo  N=37 | | | P-value of differences in resistance between treatment groups |
| --- | --- | --- | --- | --- | --- | --- | --- |
|  | Resistant  (number) | | Resistant  (%) | Resistant  (number) | | Resistant  (%) |  |
| Amoxicillin | 18 | 50 | | 8 | 22 | | 0.013 |
| Trimethoprim | 6 | 17 | | 6 | 16 | | 0.954 |
| Amoxicillin-Clavulanic acid | 6 | 17 | | 3 | 8.1 | | 0.264 |
| Ciprofloxacin | 3 | 8.3 | | 2 | 5.4 | | 0.975 |
| Pivmecillinam | 3 | 8.3 | | 1 | 2.7 | | 0.293 |
| Fosfomycin | 2 | 5.6 | | 1 | 2.7 | | 0.534 |
| Cefotaxime | 2 | 5.6 | | 1 | 2.7 | | 0.534 |
| Gentamycin | 3 | 8.3 | | 0 | 0 | | 0.074 |
| Ceftazidime | 0 | 0 | | 1 | 2.7 | | 0.321 |
| Nitrofurantoin | 1 | 2.8 | | 0 | 0 | | 0.305 |
| Meropenem | 0 | 0 | | 0 | 0 | | No resistance found |

Table S3: Antibiotics prescribed for acute urinary tract infections.

| Antibiotic prescribed | Norway  (n=95)  Number (%) | Poland  (n=15)  Number (%) | Sweden  (n=146)  Number (%) | The Netherlands  (n=143)  Number (%) | Total  (n=399)  Number (%) |
| --- | --- | --- | --- | --- | --- |
| Nitrofurantoin | 14 (15) | 3 (20) | 55 (38) | 67 (47) | 139 (35) |
| Pivmecillinam | 55 (58) | 0 (0) | 74 (51) | 0 (0) | 129 (32) |
| Fosfomycin | 0 (0) | 7 (47) | 0 (0) | 44 (31) | 51 (13) |
| Trimethoprim | 15 (16) | 0 (0) | 4 (2.7) | 11 (7.7) | 30 (7.5) |
| Ciprofloxacin | 1 (1.1) | 2 (13) | 6 (4.1) | 12 (8.4) | 21 (5.3) |
| Trimethoprim / Sulfamethoxazole | 7 (7.4) | 0 (0) | 1 (0.68) | 3 (2.1) | 11 (2.8) |
| Amoxicillin/Amoxicillin clavulanic acid | 1 (1.1) | 1 (6.7) | 1 (0.68) | 3 (1.4) | 6 (1.5) |
| Cefadroxil | 0 (0) | 0 (0) | 3 (2.1) | 0 (0) | 3 (0.75) |
| Cefuroxime | 0 (0) | 2 (13) | 0 (0) | 0 (0) | 2 (0.50) |
| Cefaclor | 0 (0) | 0 (0) | 0 (0) | 1 (0.70) | 1 (0.25) |
| Norfloxacin | 0 (0) | 0 (0) | 0 (0) | 1 (0.70) | 1 (0.25) |
| Doxycyclin | 1 (1.1) | 0 (0) | 0 (0) | 0 (0) | 1 (0.25) |
| Ciprofloxacin + singel dose Tobramycin | 0 (0) | 0 (0) | 1 (0.68) | 0 (0) | 1 (0.25) |
| Missing | 1 (1.1) | 0 (0) | 1 (0.68) | 1 (0.70) | 3 (0.75) |
